# Electrochemotherapy and inducible T cell co-stimulator (iCOS) antibody therapy: a novel combinational therapy for the enhanced treatment in cancers of poor prognosis

**DOI:** 10.1186/2051-1426-2-S3-P116

**Published:** 2014-11-06

**Authors:** Morgan O'Brien, Patrick Forde, Derek Power, Declan Soden

**Affiliations:** 1 Cork Cancer Research Centre, Cork City, Ireland; 2 Cork University Hopital, Cork City, Ireland

## 

The correlation between the level of immune involvement and immune cell infiltration within a treated tumour and the clinical outcome for patients has been well established in many solid tumour types. High numbers of tumour infiltrating lymphocytes has been associated with a significantly improved prognosis. Some of the key cell types involved are *CD3^+^*and *CD8^+ ^*T cells, B cells and APCs such as dendritic cells (DCs).

Cork Cancer Research Centre (CCRC) has long utilised an ablative cancer treatment that involves delivering a pulse of electrical energy (electroporation) directly to the tumour. This treatment results in the formation of reversible pores in the cell membrane, allowing up to a 1000 fold increase in chemotherapy drug absorption (Electrochemotherapy). A significant benefit of this non thermal ablation is the release of tumour associated antigens into the microenvironment, leading to activation of APCs and subsequent cross presentation of the antigens to circulating T and B cells. While ECT facilitates the priming of a favourable immune response, the combination of iCOS antibody, capable of stimulating T cell proliferation, causes a very robust mediated regression of treated tumours. In addition, ICOS is involved in humoral immune responses (B cell germinal center formation) and increases the production of IL-4.

Preclinical work in aggressive murine models has yielded very encouraging results. The combinational therapy has achieved cures in both colorectal (CT26) and metastatic lung (LLC) cancer cell lines. Follow up immunohistochemical staining confirmed the presence of tumour infiltrating CD3^+ ^and CD8^+ ^T cells, CD45R^+ ^B cells and CD11c^+ ^DCs, post treatment.

